# CWRML: representing crop wild relative conservation and use data in XML

**DOI:** 10.1186/1471-2105-9-116

**Published:** 2008-02-25

**Authors:** Jonathan D Moore, Shelagh P Kell, Jose M Iriondo, Brian V Ford-Lloyd, Nigel Maxted

**Affiliations:** 1 Warwick HRI, University of Warwick, Wellesbourne, Warwickshire, CV35 9EF, UK; 2 School of Biosciences, University of Birmingham, Edgbaston, Birmingham, B15 2TT, UK; 3 Area de Biodiversidad y Conservación, ESCET, Universidad Rey Juan Carlos, c/Tulipán s/n, E-28933 Móstoles, Madrid, Spain

## Abstract

**Background:**

Crop wild relatives are wild species that are closely related to crops. They are valuable as potential gene donors for crop improvement and may help to ensure food security for the future. However, they are becoming increasingly threatened in the wild and are inadequately conserved, both *in situ *and *ex situ*. Information about the conservation status and utilisation potential of crop wild relatives is diverse and dispersed, and no single agreed standard exists for representing such information; yet, this information is vital to ensure these species are effectively conserved and utilised. The European Community-funded project, European Crop Wild Relative Diversity Assessment and Conservation Forum, determined the minimum information requirements for the conservation and utilisation of crop wild relatives and created the Crop Wild Relative Information System, incorporating an eXtensible Markup Language (XML) schema to aid data sharing and exchange.

**Results:**

Crop Wild Relative Markup Language (CWRML) was developed to represent the data necessary for crop wild relative conservation and ensure that they can be effectively utilised for crop improvement. The schema partitions data into taxon-, site-, and population-specific elements, to allow for integration with other more general conservation biology schemata which may emerge as accepted standards in the future. These elements are composed of sub-elements, which are structured in order to facilitate the use of the schema in a variety of crop wild relative conservation and use contexts. Pre-existing standards for data representation in conservation biology were reviewed and incorporated into the schema as restrictions on element data contents, where appropriate.

**Conclusion:**

CWRML provides a flexible data communication format for representing *in situ *and *ex situ *conservation status of individual taxa as well as their utilisation potential. The development of the schema highlights a number of instances where additional standards-development may be valuable, particularly with regard to the representation of population-specific data and utilisation potential. As crop wild relatives are intrinsically no different to other wild plant species there is potential for the inclusion of CWRML data elements in the emerging standards for representation of biodiversity data.

## Background

Current and predicted future climate change, the spread and evolution of pests, diseases and invasive species, the requirement to reduce energy inputs to agriculture and the continuing need to feed a world population expanding exponentially, pose significant challenges to our continued ability to feed and fuel humankind. In this dynamic environment, it is likely that we will increasingly need to utilise the broader crop gene pool to ensure that crop improvement can meet these challenges [1,2]. Crop species will need to be able to thrive in a drier, warmer, and more variable climate than at present, under agricultural regimes with lower requirements for energy-rich inputs, and in an environment increasingly populated by foreign and mutated pathogenic organisms, such as insect pests, fungi and viruses [3]. Generating the information needed to enable these challenges to be met is an important focus for national, regional and global crop research.

In this context, crop wild relatives (CWR) are important as sources of genes for breeding [4]. However, CWR are themselves under threat from many of the same pressures which threaten global crop yields; such as climate change, resultant ecosystem instability, natural habitat destruction resulting from the increasing use of territory for agriculture, urbanization and other infrastructures, and from the increasing industrialisation of agriculture. CWR can potentially provide the array of genetic diversity required to counter these threats; therefore, their conservation has an important role to play in underwriting global food security [5,6]. Effective conservation and utilisation of CWR taxa is dependent on the availability of, and access to, high quality ecogeographic information about the taxa (i.e., their distribution, biology, ecology and conservation status, with respect to the environments in which they grow), as well as their *ex situ *conservation status [7,8]. Although the focus of this study is *in situ *data, the schema developed does not exclude reference to or the inclusion of *ex situ *collections data.

The collation and dissemination of information relating to CWR conservation and utilisation is challenging due to the volume and diversity of experimental and observational data. According to a recent definition of what constitutes a CWR, up to 80% of plant species [9] may be considered wild relatives of crops or other species which are of socio-economic value, including the crop species themselves. Historically, ecogeographic data have been collated in a wide variety of disparate formats (e.g., paper records, herbarium specimens, collection reports, gene bank accessions and their associated passport data), and in integrative formats, such as flora, taxonomic databases and Geographic Information Systems (GIS). *De facto *standards exist for some of the data elements which are recorded in some of these formats, but the adoption of standards is by no means universal and for some data elements, there are no widely accepted standards [8].

The eXtensible Markup Language (XML) is suited to representing biological information and is the consensus choice for dissemination and exchange of biological data in many areas [10]. Accordingly, an XML schema was developed in this project to form the basis of a syntax for formatting data on CWR for exchange and dissemination; this syntax was designated CWRML. XML notation provides a means by which disparate data can be described and represented in a standard way, in documents which can be transmitted over the web and subsequently processed by software programs, or presented intelligibly to human readers.

Recent developments in the formal representation of biodiversity data notably include the Darwin Core (DwC) [11] and Access to Biodiversity Collections Data (ABCD) [12] standards, principally designed to represent data relating to *ex situ *collections. Applications of these two standards have been reviewed elsewhere [13,14]. The Taxonomic Databases Working Group (TDWG) [15] acts as a focal point for development and integration of the standards.

The proposed DwC version 2 model provides XML schemata for the representation of information about the distribution of taxa globally, and about historical events in the sampling of biodiversity, such as collection missions. DwC also provides for discipline-specific extensions to accommodate data outside of the scope of the core schemata, which potentially could accommodate CWR-specific data. DwC has been incorporated in the Distributed Generic Information Retrieval (DiGIR) [16] protocol for biodiversity database federation. The ABCD schemata provide similar functionality and the ABCD standard has been incorporated in the Biological Collection Access Service for Europe (BioCASE) [17] database federation protocol. The emerging Global Biodiversity Information Facility (GBIF)-sponsored TDWG Access Protocol for Information Retrieval (TAPIR) [18] is an effort to provide integration of the DiGIR and BioCASE protocols, and shows promise as an emerging *de facto *standard.

CWRML was developed as part of the EC-funded project, European Crop Wild Relative Diversity Assessment and Conservation Forum (PGR Forum) [19]. The project brought together a group of taxonomic, conservation and data management experts to develop a CWR Catalogue [1], data management system [20,21] and methodologies to support the conservation and sustainable utilisation of CWR. CWRML was developed, as a language for fulfilling the data communication needs of the CWR conservation and user communities, with an emphasis on the management of *in situ *(site and population) information. Data related to *ex situ *conservation is also included in the schema; however, since other standards already exist for recording detailed collection data, CWRML was not designed to fulfil this role.

During the project, the Darwin Core version 2 standard was still in flux, the ABCD standard was nascent, and neither had emerged as a global *de facto *standard. The TAPIR protocol was not implemented in available software tools. Accordingly, rather than integrating with, or incorporating, one or other of these standards, the decision was taken to implement CWRML with internal divisions broadly in line with the divisions within both the emerging Darwin Core 2 and ABCD standards. It was anticipated that this approach would facilitate integration of the CWR-specific elements of CWRML with the taxonomic- and location-specific elements of the emerging *de facto *standard at a later date.

Thus, given the diversity of the data required to support effective CWR conservation and utilisation, the lack of a currently accepted standard for representing such data, and the requirement to disseminate and present such data in an integrated form, we propose CWRML as a common language for representing and disseminating data relevant to CWR conservation and utilisation.

## Implementation

For the purpose of CWRML, we attempted to include the essential information that is required to support the conservation and sustainable utilisation of CWR. We aimed to use meaningful syntax for the naming of data types and elements that is familiar to plant genetic resource (PGR) specialists, in order that the language should be self-explanatory and human- as well as machine-readable. Similarly, we incorporated accepted standards for coding biodiversity information wherever possible, by implementing the standards as restrictions on the content of data elements and providing references to the relevant standards as comments within the schema. Where such standards contained a relatively small number of options, we enumerated the options within the restriction in full; where the options were too numerous to be enumerated here, a more generic restriction was used.

In particular, we incorporated data coding standards published by the IUCN and those accepted by TDWG, wherever possible. IUCN publishes authority files [22-24] for use in the Species Information Service (SIS) [25], an information management tool for species-related data that is under development, and in the application of the Red List Categories and Criteria [26]. TDWG standards for the syntax of taxon nomenclatural data [27] and for economic botany [28] are both widely accepted, although some amendments to the economic botany standards for CWR have been proposed [29].

The standards used were not generally available in published XML schemata, so were incorporated directly in the CWRML schema for simplicity of implementation. However, it was recognised that in future, it might be more appropriate to isolate each standard in its own XML schema, which would then ideally be further developed by the body concerned with defining the standard in question. Given recent developments in the representation of biodiversity conservation data (see Discussion below), this situation is likely to be brought to fruition in due course.

We aimed to use a sufficient minimum of markup to represent the data unambiguously. In these guidelines, we propose the following structure for the XML application, CWRML. We decided to define all tags as elements without attributes. In our view, additional clarification of the units of information represented here is not warranted.

## Results

### Structure and syntax of CWRML documents

The full XML schema describing the structure and syntax of a CWRML document is included with this paper [see Additional file 1], so is described here in outline only and to highlight references to external syntactic authorities. Details of the data standards used within the schema are also summarised [see Additional file 2].

**Figure 1:**
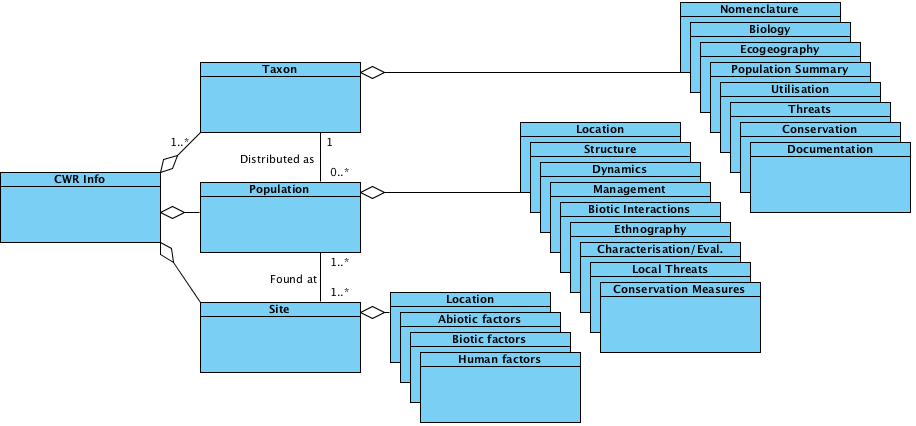
**Universal Modelling Language (UML) class diagram showing the relationships between the main classes of data comprising a CWRML document**. The diamond symbol at the end of a line indicates that the class at that end of the line aggregates data from the class(es) at the other end of the line. The small number at the end of a line indicates the allowed number of instances of the class at that end of the line per instance of the class at the other end of the line; 0..* indicates zero or more, 1..* indicates one or more.

A CWRML document consists of data in three main entities identified as categories of data required for CWR conservation management and utilisation potential: the taxon under consideration, the sites at which the taxon occurs, and the populations of the taxon at individual sites [30]. Figure 1 shows the main classes of data that comprise a CWRML document. The taxon information section contains data fields for describing information about an individual CWR taxon and optional summary information about the worldwide occurrence of the taxon. This section also includes the identification of the crop to which the taxon is related and the degree of relationship [31]. The site information section contains a sequence of site elements, each containing a sequence of elements describing features of a specific geographical location, which are relevant to conservation and utilisation of CWR taxa extant at that site and for *ex situ *collection management. The population information section contains a sequence of population elements, each describing the information which is relevant to the conservation and utilisation of a particular geographically located population of a particular CWR taxon. Elements are further subdivided into sub-elements, where appropriate, to provide for logical subsets of the individual data elements applicable in different contexts in which CWRML might be used.

Sites and populations are both spatially located, but there is considerable debate within the CWR research community about the definitions and scope of 'sites' and 'populations', and whether a site may contain several populations, and/or a population span several sites [30]. Accordingly, CWRML does not constrain sites and populations relative to each other, but allows both to be assigned, independently, to spatial locations. The scope and extent of a spatial location is not constrained within the schema and could be a point location (latitude and longitude coordinates), a region, a country, or an arbitrary spatial polygon defined by geo-location coordinates.

### Taxon information

Taxon information is divided into eight constitutive elements. The Nomenclature element describes formal nomenclature delimiting the taxon and uses the TDWG syntax. The Biology element describes the biology of the taxon, pertinent to CWR conservation and utilisation. Restrictions on the content of data elements are used for the biological data, including the standard descriptions of life form [32]. The Ecogeography element describes the ecogeographic 'envelope' within which the taxon occurs worldwide [7]. Restrictions are used in some of the ecogeographic data elements, including the IUCN standards for extent of occurrence and area of occupancy [26] and the FAO standard for soil type [33]. Two alternative standards are included for habitat classifications: the IUCN global standard [22] and the European Nature Information System (EUNIS) habitat types [34]. Both standards are widely used and the EUNIS classification provides for greater accuracy in habitat description. The application of the EUNIS standard is only appropriate in a European context; however, other regional standards may be appended to the schema in future, if more detailed description is required than currently supplied by the IUCN global standard [22]. The Population Summary element describes the worldwide distribution and occurrence of the taxon. The Utilisation element describes the ways in which the taxon is utilised and its potential for utilisation in crop breeding programmes. The TDWG Economic Botany standard [28] is used for describing the uses and ethnobotany of taxa. Where data are available, the Gene Pool concept [35] is used to describe the degree of relationship between the CWR and the crops to which they are related; otherwise, the Taxon Group concept [31] is used. The Threats element describes any threats to the survival of the taxon in the wild, using the IUCN standard [23], and whether these are the subject of a formal Red List assessment. The Conservation element uses the IUCN standard syntax [24] to describe the worldwide conservation status of the taxon and any actions currently being taken to conserve it, both *in situ *and *ex situ*. The Documentation element describes the information relating to the reference sample of the taxon (e.g., the herbarium voucher specimen, illustration or photograph).

### Site information

The site information type describes the ecogeography of a site at which the CWR taxon is found, including the spatial location, microclimate, geomorphology, geology, soil, vegetation and human interactions at the site. Again, both EUNIS [34] and IUCN [22] standards for habitat classification are available as distinct elements.

### Population information

This element describes the location, size, structure, dynamics, management, biotic interactions, ethnography, characterisation and evaluation, local threats and conservation measures, relating to an individual population of a CWR taxon. Some of the available standards that can be used to describe populations are the IUCN standard [23] for the category of threat to an individual population, the Braun-Blanquet scale [36], which can be used in the description of the target population size and the associated vegetation, and the Moss and Guarino method [37], which can be used to categorise spatial patterns of individuals. Population trends are described following the IUCN Red List Criteria [26] in order to feed directly into the Red Listing process.

## Discussion

The TDWG DwC XML schema [10] is intended as a collection of core XML schemata for the representation of information related to *ex situ *biodiversity collections. Version 2 of the Darwin core is currently being developed by TDWG, in a collaborative project. The proposed Darwin core version 2 contains generic sections, such as those describing the information about geographic location, occurrence, and some site information, which overlap with some of the elements presented here. When work on this version of Darwin core is completed, sections of CWRML specific to CWR conservation and utilisation may be extracted from the schema and proposed as a CWR extension to the Darwin core. This would accord with best practice in schema design, by decomposing a complex schema with a wide scope (such as CWRML) into a set of interoperable, independent, reusable schemata, and by reusing existing schemata. A similar approach might also be taken to integration with the ABCD schemata [12].

CWRML incorporates a number of restrictions relating to coding of observational data, but a number of additional restrictions might also be applied to future versions. For example, data elements such as grazing pressure could be restricted and standards are available for describing dominant and associated vegetation types.

In April, 2007, IUCN and the Organisation for Advancement of Structured Information Systems (OASIS) agreed to form a Biodiversity Conservation Member Section under OASIS, and develop a programme for this group [38]. It is anticipated that this group will bring forward data standards for conservation biology, including species conservation status, management effectiveness categories and ontology for protected areas, priority conservation site descriptors and status, as well as common vocabularies for conservation action and best practice and geospatial data standards. The group seeks to incorporate existing innovations in their work; as there is intrinsically no difference between CWR and other wild plant species [39], the CWRML may make a useful contribution to this standards-development process.

The need for gathering data concerning the conservation and utilisation of CWR in a systematic and standardised way is so fundamental that other international projects linked to CWR (e.g., the GEF-funded project "In situ Conservation of Crop Wild Relatives through enhanced information management and field application" [40,41]) are now also working on developing data standards in this field. CWRML has also been made available to this project to facilitate standardisation in a coordinated way.

## Conclusion

CWRML provides a compact representation for CWR conservation and utilisation data that can be delivered by a web server as self-describing documents, which are machine-readable and intelligible to human readers. It was developed in collaboration with conservation biologists and practitioners, and tested using case studies of disparate CWR taxa, and so is likely to have broad applicability in these fields. Applications include representing and communicating data in the context of IUCN Red List assessments, conservation status assessments, gap analysis, describing utilisation potential and country-wide, crop-specific, or wild taxon-specific genetic resources distribution. The language provides a ready platform for developing integrative infrastructure, allowing databases which implement the language to provide data to web-service-based federated queries.

CWRML contains a number of data elements with corresponding syntactic standards, which may make valuable additions or contributions if integrated with emerging standards for the representation of biodiversity conservation data.

The development of CWRML brought to light a number of gaps in current syntactic standards in the representation of biodiversity and conservation biology data. In particular, the sub-elements in the Population data element were generally found to lack formal syntax, and further standards-development in this area would be fruitful in allowing such data sets to be integrated and queried.

## Availability and Requirements

**Project name: **PGR Forum

• **Project home page: **

• **Operating system(s): **Platform independent

• **Programming language: **XML

• **Other requirements: **None

• **License: **GNU GPL

• **Any restrictions to use by non-academics: **None.

## Authors' contributions

JMI, NM and SPK contributed to the development of the data syntax, and standards used. NM, BVF-L, SPK and JDM proposed the original relational model for CWR information. SPK and JDM revised the relational model in the light of user-workshops and user-testing against a number of case studies. JDM devised the schema and wrote the manuscript. All authors contributed to the final manuscript.

## Supplementary Material

Additional file 1 Crop Wild Relative Markup Language XML Schema. Click here for file

Additional file 2 A spreadsheet listing acceptable values for individual data elements and references to external standards. Click here for file
